# Sirtuin 3 Plays a Critical Role in the Antidepressant- and Anxiolytic-like Effects of Kaempferol

**DOI:** 10.3390/antiox11101886

**Published:** 2022-09-23

**Authors:** Hao-Yuan Li, Jing Wang, Ling-Feng Liang, Shi-Yu Shen, Wei Li, Xiao-Rong Chen, Bing Li, Yu-Qiu Zhang, Jin Yu

**Affiliations:** 1 Department of Integrative Medicine and Neurobiology, School of Basic Medical Sciences, Shanghai Medical College, Fudan University, Shanghai 200032, China; 2 Center Laboratories, Jinshan Hospital of Fudan University, Shanghai 201508, China; 3 State Key Laboratory of Medical Neurobiology and MOE Frontiers Center for Brain Science, Institutes of Brain Science, Shanghai Medical College, Fudan University, Shanghai 200032, China; 4 Shanghai Key Laboratory of Acupuncture Mechanism and Acupoint Function, Fudan University, Shanghai 200433, China

**Keywords:** kaempferol, menopausal depression, antioxidant effects, superoxide dismutase 2, Sirtuin3

## Abstract

An estimated 20% of women experience depression at some point during menopause. Hormone replacement therapy (HRT), as the main therapy for depression and other menopausal syndromes, comes with a few undesirable side effects and a potential increase in cancer and cardiovascular risk. Consequently, there is a dire need for the development of new therapies to treat menopausal depression. Oxidative stress combined with the decline in sex hormones might explain the occurrence of psychological symptoms characteristic of menopause. Therefore, antioxidants have been suggested as a promising therapy for aging-associated diseases, such as menopausal depression. As a flavonoid antioxidant, kaempferol might have a potential neuroprotective action. Hence, the study was conducted to assess the potential antidepressant action of kaempferol and clarify the underlying mechanism. The results show that kaempferol has potential beneficial effects on VCD-induced rodent model of menopausal depression and produces antioxidant effects as well as increases the deacetylation of superoxide dismutase 2 (SOD2) and the protein level of Sirtuin3 (Sirt3) in the hippocampus. On the contrary, *Sirt3* depletion abrogated the antidepressant- and anxiolytic-like effects as well as antioxidant effects of kaempferol. In conclusion, kaempferol might produce antidepressant effects via upregulating the expression of Sirt3, the major deacetylase in mitochondria, and subsequently activate the mitochondrial antioxidases. These findings shed some light on the use of kaempferol or vegetables and herbs that contain kaempferol as a complementary therapy for menopausal depression.

## 1. Introduction

Depression is the most frequent symptom in menopausal women, and is even a major cause of medical consultation [1,2]. The process of menopause is characterized by a gradual decrease in sex hormones, including estrogen and progesterone [3]. In addition, the neuroprotective effects of estrogen have been verified widely [4]. The constant changes and fluctuation of hormones (estrogen, progesterone) that occur during menopause is thought to increase vulnerability to mood disorders [3]. Therefore, hormone replacement therapy (HRT) has emerged and has been routinely prescribed for menopausal women to relieve menopausal symptoms, including depression. However, after years of application and research, scientists noticed the mixed results of HRT and have thus adjusted its indications [5,6]. Therefore, there is an enormous unmet need for the development of alternative or new therapies to treat menopausal depression.

Another major type of depression in women that resembles menopausal depression is postpartum depression, which is also thought to be the result of a rapid decline in reproductive hormone levels that occurs immediately after delivery [7]. Moreover, a substantial amount of literature has suggested the close link between oxidative stress and depression, especially menopausal depression [8]. Therefore, in addition to declining hormone levels, aging-related oxidative stress might be a major cause of depression during menopause. As we know, oxidative stress is an important aspect of the aging process [9]. It is caused by the overproduction of reactive oxygen species (ROS) and other free radicals, which disrupt the body’s antioxidant defense system [9]. Antioxidant levels decline as the body ages, and the human body is susceptible to various age-related pathologies. Therefore, supplementation with antioxidants has been proposed as a promising therapy for aging-associated diseases, such as menopausal depression [10,11].

Many natural compounds have been considered as natural antioxidants, such as kaempferol. Kaempferol, a flavonoid antioxidant present in fruits, vegetables [12] and simultaneously a key component in many traditional Chinese medicines for depression such as Xiaoyaoshan, *Hemerocallis fulva* L. and so on [13,14,15,16]. However, its potential beneficial effects on menopausal depression still have not been clarified.

In vitro, kaempferol acts as a scavenger for superoxide radicals and free radicals. In vivo, it acts as antioxidants and has anti-inflammatory properties [17,18], which underlie its neuroprotective effects in central nervous system diseases [16].

Our previous studies have confirmed that mitochondrial functions, such as membrane potential, superoxide dismutase (SOD) activity and total antioxidant capacity, were significantly declined in the hippocampus from aged female rats or ovariectomized (OVX) rats [19,20]. In this study, we prefer to further investigate the influence of kaempferol on mitochondrial function, especially on SOD activity. As we know, SOD2 is the main SOD in mitochondria, which scavenges ROS only while it was deacetylated. Meanwhile, Sirtuin 3 (Sirt3), as a major deacetylase in mitochondria is responsible for deacetylation of mitochondrial proteins, including SOD2 [21]. Interestingly, a recent study in vitro has hinted that kaempferol can promote the expression of Sirt3 [22].

Therefore, this study aimed to assess the potential beneficial effects of kaemperol on menopausal depression and explore the molecular targets which mediate its potential antidepressant effects.

## 2. Materials and Methods

### 2.1. Animals

Female 129S1/SvImJ mice (8–10 weeks old) were purchased from Shanghai Model Organisms Center, Inc., Shanghai, China, (SMOC). Female C57BL/6J mice (8–10 weeks old) were purchased from Shanghai SLAC Laboratory Animal Co. Ltd., Shanghai, China, *Sirt3* knockout mice (*Sirt3*^−/−^ mice) were kindly supplied by Prof. Wei Yu (Fudan University, Shanghai, China) [23]. All animals were bred and placed in climate-controlled rooms (22 ± 1 °C) with 12:12-h light-dark cycle (light period starts at 7 a.m.) and food and water available *ad libitum*. All animal experiments were approved by the Experimental Animal Ethics Committee of Shanghai Medical College, Fudan University, Shanghai, China (20180302-086) and performed in accordance with the Guide for the Care and Use of Laboratory Animals.

### 2.2. VCD Induced Ovary Failure Modelling [24,25]

**Figure 1:**
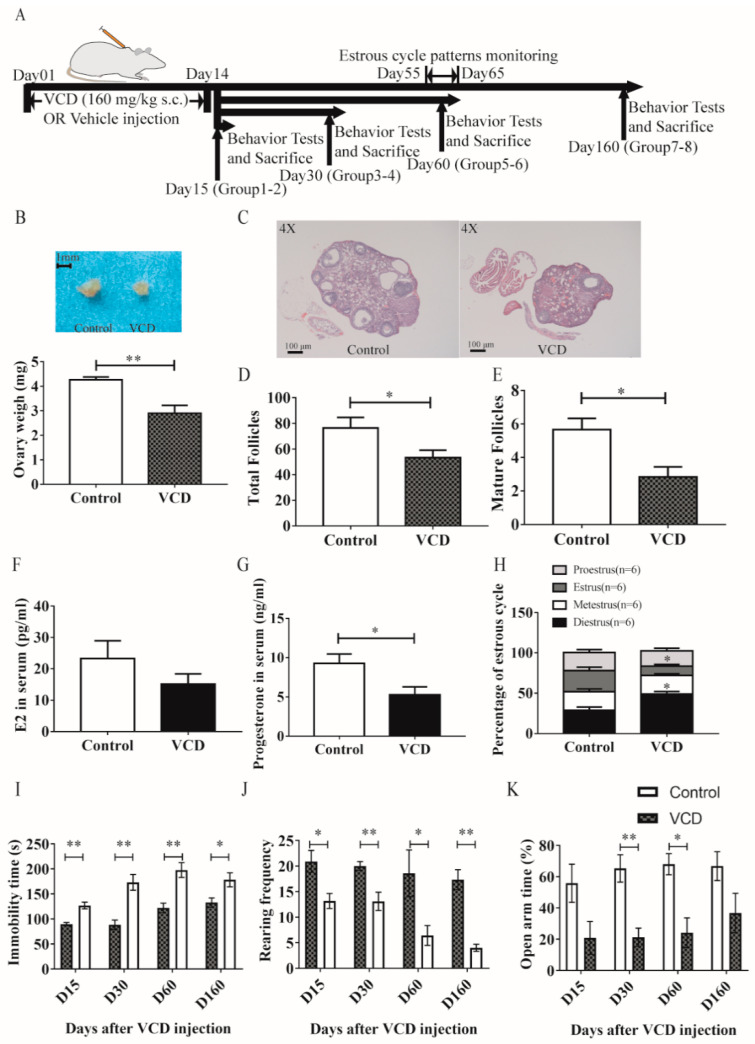
VCD exposure induces ovarian damage, depression- and anxiety-like behaviors in female mice. (**A**) Schematic of the protocol for VCD exposure, estrous cycle patterns monitoring and behavioral testing. (**B**) Gross morphology (upper panel) and weight (low panel) of mice ovaries. (**C**) HE staining of ovaries at 4× for control and VCD-treated mice. (**D**,**E**) Total ovarian follicle numbers (**D**) and mature ovarian follicle numbers (**E**) in the ovaries of control and VCD-treated mice. (**F**,**G**). Serum concentrations of estradiol (pg/mL) (**F**) and progesterone (ng/mL) (**G**) in control and VCD-treated mice. (**H**) Percentage of each phase of the estrous cycle in control and VCD-treated mice. Pearson’s chi-square test were used for comparison each phase between the two groups. (**I**). Immobility time in FST. (**J**) Rearing frequency in OFT. (**K**) Percentage of time spent in the open arms in EPM. Statistical significance in (**B**–**G**) was determined using Mann–Whitney test or Students’ *t*-test. Statistical significance in (**I**–**K**) was determined using two-way ANOVA analysis with Sidak’s multiple comparison test or Mann–Whitney test. Data are represented as mean ± SEM, *n* = 6 per group; * *p* < 0.05, ** *p* < 0.01.

Mice received a subcutaneous injection of VCD (4-Vinylcyclohexene Diepoxide, 160 mg/kg, 0.2 mL, dissolved in corn oil, Sigma, St. Louis, MO, USA, #94956) once a day for 14 days to build the ovary failure model (Figure 1A). The day that mice started with VCD treatment in all experiments is exhibited in Table S1. Control mice were administered with corn oil (vehicle). VCD induces the selective damage of primary and primordial follicles in the ovary to accelerate atresia, but leaves the rest of the ovary intact to produce residual hormones. Vaginal cytology was utilized to determine the absence of estrous cycles that indicates ovarian failure. All the injected mice were free of adverse symptoms and infections and recovered well.

### 2.3. Estrous Cycle Examination

Vaginal cytology was used to monitor and classify the stage of estrous cycle as described previously [26]. Stages of the estrous cycle were identified using the relative ratio of cell types, such as leukocytes, cornified epithelial cells and nucleated epithelial cells observed in smears [27]. In detail, during proestrus, cells are almost entirely clusters of round nucleated epithelial cells with resemblance in appearance and size. During estrus, keratinized squamous epithelial cells predominate and present in densely packed clusters. During metestrus, cells are mainly small, darkly stained leukocytes, with some keratinized squamous epithelial cells in fragments. During diestrus, leukocytes still predominate; meanwhile, rare cornified squamous epithelial cells may remain. Metestrus can be distinguished from diestrus by the appearance of nucleated epithelial cells in diestrus. Estrus cycle disturbance is a prominent feature of ovarian failure.

### 2.4. Ovarian Embedding and H&E Staining

The ovaries were obtained from the mice and the surrounding adipose tissue was removed. Then, the ovaries from the mice were fixed by immersion in 4% paraformaldehyde for one day. After fixation, the ovaries were dehydrated in increasing the concentration of alcohol (75%, 85%, 95% and 100%). For embedding, the dehydrated ovaries were soaked in xylene followed by paraffin. After solidification, the embedded ovaries were serially sectioned (3 µm thick sections). Then, the sections of the ovaries were dewaxed in xylene and rehydrated in decreasing concentration of ethanol before staining. For HE staining, the sections were incubated in hematoxylin for 10 min and differentiation solution for 15 s followed by rinse with flowing distilled water. Next, the sections were soaked in distilled water for 15 min and stained with eosin for 2 min followed by rinse and soak with distilled water. Finally, the sections were dehydrated in increasing concentration of alcohol and cleared in xylene before being sealed for observation.

### 2.5. Follicle Counts

All the HE-stained ovarian slides were scanned at 10x using an Olympus microscope, and the number of total follicles and mature follicles were manually counted. Follicles were identified as: (a) primordial: an oocyte surrounded by a monolayer of granulosa cells; (b) primary: a fully grown oocyte surrounded by zona pellucida and granulosa cells; (c) preantral: two or more layers of granulosa cells surrounded by early theca; (d) early antral: an antrum visible, with at least two layers of cuboidal granulosa cells; (e) mature: a conspicuous large antrum cavity, with few layers of granulosa cells. We distinguish mature follicles from other follicles mainly based on cavity size and follicle size.

### 2.6. Radioimmunoassay of Estradiol and Progesterone

The blood (~500 μL) was obtained from orbital sinus after animal behavioral tests before being sacrificed. After anesthesia, the eyeballs of mice were quickly removed with tissue forceps and the blood from the orbital sinus was dropped into 1.5 mL EP tubes, then the mice were sacrificed by cervical dislocation under anesthesia [28]. The whole blood was centrifuged for 20 min (3000 rpm, 4 °C). The supernatant (serum) was obtained and stored in 1.5 mL EP tube at −80 °C. The estradiol and progesterone concentration of the sample serum was measured by an estradiol and progesterone radioimmunoassay kit (Beijing North Institute of Biotechnology, Beijing, China) under the guidance of the experimental operation manual. Marker antibody was added to the serum samples and standard samples for incubation for 2 h. Next, the separated agent was added and incubation for 15 min, then the sample was centrifuged at 3800 rpm for 15 min. The radioactivity was measured by a Xi’an Nuclear Instrument Factory XH6080 Radioimmunoanalyzer. The concentration was determined according to the radioactivity. In brief, the desired reading of standard S0 was B_0_, the reading of each standard tube or sample tube was B, while the reading of the nonspecific tube was NSB, and the percentage binding rate could be calculated by formula B/B_0_ = (B − NSB)/(B_0_ − NSB) ∗ 100%. The logit value of each standard point or sample tube could be calculated by formula logit = ln[(B/B_0_)/(1 − B/B_0_)]. Based on the above results, we drew a linear fitting line with standard concentrations as abscissa and the logit value as ordinate, which could provide the corresponding concentration value according to the B/B_0_ of the sample to be tested.

### 2.7. Kaempferol Treatment

**Figure 2:**
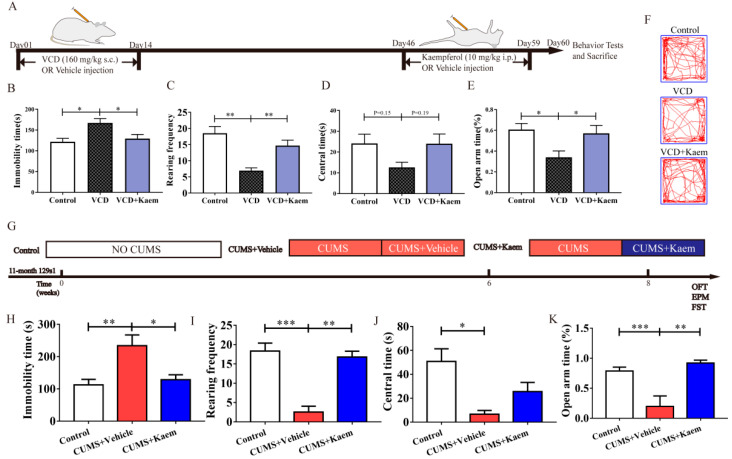
Kaempferol exhibits antidepressant- and anxiolytic-like effects in two mice models of menopausal depression. (**A**) Schematic of the protocol for VCD exposure and kaempferol treatment in mice. (*n* = 10–13 per group) (**B**) Immobility time in FST. (**C**,**D**). Rearing frequency (**C**) and time spent in the central area (**D**) in OFT. (**E**) Percentage of time spent in the open arms in EPM. (**F**) Representative activity traces in OFT. (**G**) Schematic of the protocol for CUMS and kaempferol treatment (*n* = 4–5 per group). (**H**) Immobility time in FST. (**I**,**J**) Rearing frequency (**I**) and time spent in the central area (**J**) in OFT. (**K**) Percentage of time spent in the open arms in EPM. Data are graphed as mean ± SEM, one-way ANOVA analysis with Tukey’s multiple comparisons test or Kruskal–Wallis test with Dunn’s multiple comparisons test: * *p* < 0.05, ** *p* < 0.01, *** *p* < 0.001.

Female 129S1 *Sirt3*^+/+^ or *Sirt3*^−/−^ mice with successful premature ovarian failure modeling received further kaempferol treatment. Kaempferol (SELLECK, S2314) was dissolved in DMSO to prepare a stock solution of 50 mg/mL, and then it was diluted with normal saline so that the DMSO concentration was 0.72% *v*/*v*, while the kaempferol concentration was 0.36 mg/mL. The kaempferol-treated mice were injected with kaempferol (10 mg/kg, i.p.) from day 46 for 14 days, while the control mice were injected with an equal volume of 0.72% *v*/*v* DMSO/NS solution at the same time. After the end of the kaempferol treatment (day 60 from VCD modeling starting), the behavioral tests were performed, and then, the hippocampus/ovary/blood were collected. The procedure of study was exhibited in Figure 2A and Table S1.

### 2.8. Behavioral Tests

#### 2.8.1. Forced Swimming Test (FST)

The FST apparatus consists of a Plexiglas cylinder 30 cm high and 15 cm in diameter. Before the test, the cylinders were filled with water (23 ± 2 °C) to a depth of 15 cm, which was deep enough that the mouse could not touch the bottom of the cylinder with its tail. The water was replaced between subjects. Based on previous studies [29], the mice were placed in the apparatus and behavior was monitored for 5 min. The total immobility time in the test was measured and counted by SuperMaze software (Version 3.01, Shanghai Xinruan Information Technology, Shanghai, China).

#### 2.8.2. Open Field Test (OFT)

The OFT was utilized to characterize locomotor activity and anxiety of mice in a novel environment. Testing was performed in a dimly open-field arena (50 cm × 50 cm × 40 cm, 40 lux) in order to minimize anxiety effects on locomotion. All mice were placed into the center area of open field at the beginning of the trial. The rearing frequency was counted when the subject animals were stranded on both hind paws in a vertical upright position. The distance traveled within 5 min was recorded and analyzed with the VisuTrack animal behavior video analysis system (Shanghai Xinruan Information Technology, Shanghai, China).

#### 2.8.3. Elevated Plus Maze Test (EPM)

The EPM apparatus consists of two open arms (30 × 5 cm) and two closed arms (30 × 25 × 5 cm) perpendicularly crossed. The arms are connected to one another by a central platform (5 × 5 cm). The mazes are elevated 40 cm above the ground. The mice were placed in the central platform heading to the open arm when starting the 5-min test session. The time spent in the open and closed arms within 5 min was also recorded and assessed with the VisuTrack animal behavior video analysis system as mentioned above.

### 2.9. Superoxide Dismutase (SOD) Activity

The pre-isolated mitochondrial of hippocampal samples were preserved in mitochondrial storage buffer (Beyotime, Shanghai, China, C3606) on ice. SOD activity of the hippocampal samples was evaluated using a Sigma SOD assay kit (Sigma 19160) according to the manufacturer’s instructions. In brief, 20 μL of hippocampal samples in Tris-Cl buffer pH 7.5 was added to a well of a 96-well plate and mixed with 200 μL of WST-1 (2-(4-iodophenyl)-3-(4-nitrophenyl)-5-(2,4-disulfophenyl)-2H-tetrazolium, monosodium salt), which was used as an indicator dye. Xanthine/xanthine oxidase (0.25 U) reaction produces superoxide radicals. Hence, the addition of 20 μL of xanthine oxidase solution initiates the reaction. After incubating the plate at 37 °C for 20 min, the absorbance at 450 nm was measured using a microplate reader (SpectraMax Paradigm Multi-Mode Microplate Reader, Molecular Devices, San Jose, CA, USA). The enzymatic activity was calculated per 1 mL of sample and expressed in international units (U).

### 2.10. Mitochondrial Membrane Potential Test

The mitochondrial membrane potential (ΔΨm) was assessed using JC-1(Invitrogen, Carlsbad, CA, USA). JC-1 is a cationic dye (green) that exhibits potential-dependent accumulation in mitochondria. In mitochondria, JC-1 starts forming reversible complexes called J aggregates in a concentration-dependent manner. J aggregates show excitation and emission in a red spectrum (maximum at ~590 nm) rather than green (maximum at ~530 nm). Previous studies have shown a linear relationship between the fluorescent ratio (530 nm/590 nm) of JC-1 and membrane potential in a physiological range [30]. Moreover, in a variety of cell types, as well as in intact tissues and isolated mitochondria, JC-1 dye is useful as an indicator of mitochondrial membrane potential.

Mitochondria were isolated from hippocampal tissues by mechanical homogenization and differential centrifugation using a mitochondria isolation kit for tissue (Abcam, Cambridge, UK, ab110168) with published protocols [31].

After isolation, mitochondria were resuspended in a minimal amount of storage buffer, and incubated in the dark for 30 min at 37 °C with pre-diluted JC-1(JC-1: DMSO = 1:10) and reaction buffer (Reaction buffer: pre-diluted mitochondria: pre-diluted JC-1 = 1900:100:2). Finally, the plate reader (SpectraMax Paradigm Multi-Mode Microplate Reader, Molecular Devices) was utilized to measure the fluorescent intensities for both J-aggregates and monomeric forms of JC-1 at Ex/Em = 490/525 nm and 530/590 nm respectively.

### 2.11. Total Antioxidant Capacity (TAC)

TAC was detected with the total antioxidant capacity assay kit (MAK187, Sigma-Aldrich, St. Louis, MI, USA). In brief, the total antioxidant capacity of the sample was approximated by testing the ability of the sample to reduce Cu^2+^ to Cu^+^ and was presented by the Cu^+^ colorimetric probe. In the test, the antioxidant capacity of the substrate was indicated by the discoloration of the chelate compound. Trolox (Water-soluble vitamin E) 1 mM solution was utilized as a standard and serial dilution were further prepared (0, 4, 8, 12, 16 and 20 nmol/well of Trolox standard solution) Cu^2+^ Working Solution was made by mixing Assay Diluent and Cu^2+^ Reagent at a ratio of 50:1. Then, 100 μL of a test sample or standard solution was added to each well, followed by 100 μL Cu^2+^ Working Solution. The plate was incubated at room temperature for 90 min to complete the reduction reaction. Finally, the plate reader (SpectraMax Paradigm Multi-Mode Microplate Reader, Molecular Devices) was utilized to detect the absorbance at 570 nm. The relative TAC of the sample was calculated from standard curve using Trolox.

### 2.12. Protein Isolation and Western Blotting

The hippocampi were separated from the overlying cortex and lysed with RIPA lysis buffer containing protease–phosphatase inhibitor cocktail (Sigma-Aldrich, P8340), which was added according to tissue weight (100ul:1g). The lysates were treated with Sonifier (Hielscher, Teltow, Germany, UP50H), followed by centrifugation at 12,000 rpm at 4 °C for 20 min and the supernatant was collected as a total protein sample. The BCA protein assay kit (Thermo Scientific, Waltham, MA, USA, 23227) was utilized to quantify the total protein contents of the samples. After diluted to the same protein concentration with water, the samples were heated with loading buffer at 100 °C for 10 min. The samples were then separated by SDS-PAGE electrophoresis and transferred to polyvinylidene difluoride (PVDF) membranes (Millipore, Burlington, MA, USA, IPVH00010) at 4 °C. After blocking with 1% bovine serum albumin or 5% skim milk, the membranes were incubated with primary antibody (Ac-K68-SOD2: Abcam, ab137037; SOD2: CST, 13141; Sirt3: CST, 2627; VDAC: Abcam, ab14734) overnight at 4 °C in a shaking incubator. After incubation, membranes were washed with 0.1% TBST three times, 10 min each time, then incubated with HRP-conjugated secondary antibody (Beyotime, A0208) for 2 h at room temperature, washed with TBST three times as before and bands were visualized using Pierce ECL Western blotting substrate (Thermo Scientific, # 32106) and analyzed using ImageJ software (Version 1.48, NIH, Bethesda, MD, USA).

### 2.13. Nuclear Protein Isolation and Western Blotting

The nuclear protein was isolated with a nuclear and cytoplasmic protein extraction kit (P0028, Beyotime). In brief, by using cytoplasmic protein extraction reagents A and B, the homogenized hippocampus cells were fully swollen under low osmotic pressure conditions, and then the cell membranes were disrupted to release cytoplasmic proteins. The nuclei were pelleted by centrifugation. Finally, the nuclear protein was extracted by high-salt nuclear protein extraction reagent. Western Blotting was performed as mentioned above. The isolated nuclei and cytoplasmic protein samples were separated by electrophoresis on SDS-PAGE and transferred to polyvinylidene difluoride membranes. The membranes were incubated with primary antibody overnight at 4 °C in a shaking incubator. The primary antibodies used in this part are Histone H3 antibody (1:1000, 9715S, CST), ERRα (E1G1J) Rabbit mAb (1:1000, 13826, CST), and β-actin (1:10000, HRP-60008 Proteintech).

### 2.14. Chronic Unpredictable Mild Stress (CUMS)

The CUMS model has been widely used to induce depression in male adult rodents. It also can be employed in the study of menopausal depression. Ovariectomy combined with CUMS [32,33] and ovariectomy combined with maternal separation and CUMS [34] have been used to mimic menopausal depression in animal models, but ovariectomy alone has also been shown to induce depression-like behaviors [35]. Moreover, the CUMS-induced animal model of depression has been successfully established in our lab for several years [36,37]. So, to further confirm the antidepressant action of kaempferol in menopausal depression, we established a mice model of menopausal depression using aged female mice exposed to CUMS. The CUMS procedure was adapted from our previous publication [36] by removing heating (45 °C, 5 min) and exposure from a predator for 15 min, which can cause physical injury in aged mice. At the same time, we added a stressor of food or water deprivation (24 h), and increased the shaking time to 8 h as a strong stressor. In brief, mice (11 months old, 129s1 female mice) were given two stressors a day for eight weeks. The stressors were as follows: (1) fasting food and water for 24 h, (2) confinement for 30 min, (3) wet bedding for 8 h, (4) forced swimming at 4 °C for 5 min, (5) shaken with cage for 8 h, (6) white light strobe stimulation for 8 h, (7) noise stimulation for 8 h. In the last 2 weeks of CUMS exposure, aged mice in the kaempferol treatment group were simultaneously treated with kaempferol treatment (details same as Section 2.7) for 2 weeks, while aged mice in vehicle group treated with DMSO/NS solution (same as 2.7). Meanwhile, the control mice were naïve and unexposed to CUMS for 8 weeks (Figure 2G, Table S1).

### 2.15. Overexpression of Sirt3

The overexpression target gene is NM_022433. The virus vector used was pAAV-SYN-Sirt3-P2A-EGFP-3FLAG, while the control virus vector was pAAV-SYN-MCS-EGFP-3FLAG. These viruses were purchased from Heyuan Biotechnology (Shanghai, China). Female C57BL/6J mice with disordered estrous cycle/prolonged estrus after VCD modeling were selected for virus injection. After anesthesia with sodium pentobarbital, the mice were fixed in a stereotaxic apparatus. Virus injection coordinates are (front fontanelle as the origin of coordinates): AP = −2.0 mm, ML = ±1.5 mm, DV = 2.0 mm. These serotype AAV2/9 viruses were injected at a titer of 5.00 E + 13V.G./mL, 0.5 μL each hippocampus. The follow-up behavioral experiments were carried out two weeks after the injection when the virus was stably expressed.

### 2.16. Statistical Analysis

All results in this experiment are shown as mean ± standard deviation (MEAN ± SEM). GraphPad Prism 7.0 (GraphPad Software, San Diego, CA, USA) was utilized for the statistical analysis. First, Shapiro-Wilk test were used for the assessment of normality and F test, Bartlett test or Brown–Forsythe test for homoscedasticity. Then according to whether the data is normally distributed and whether the variance is homogenous, we choose the corresponding statistical method. If the data are normally distributed and homogeneous of variance, two-tailed Student’s t-test; if not, the Mann–Whitney test was utilized to compare the data between the two groups. One-way ANOVA with Tukey’s multiple comparisons test or Kruskal–Wallis test with Dunn’s multiple comparisons test were utilized for comparing multiple groups. Two-way ANOVA with Tukey’s multiple comparisons test or Mann–Whitney test were utilized for multivariate data comparison. Pearson’s chi-square test was utilized for the estrous cycle related data analysis in Figure 1E. The statistical significance was set as *p* < 0.05.

## 3. Results

### 3.1. VCD Exposure Induces Ovarian Damage, Depression- and Anxiety-like Behaviors in Female Mice (Figure 1)

In order to explore the potential antidepressant effects of kaempferol, we first established a mice model of menopausal depression (Figure 1A). Chronic exposure to 4-vinylcycloxene diepoxide (VCD) [38] in mice [39] can model perimenopause in women. However, seldom depression- and anxiety-like phenotypes were detected in the model. According to the previous studies [24,39], we assessed ovarian status and function on day 60 after chronic exposure to VCD (sc 160 mg/kg, qod, 14 days, Day 1 to Day 14, Figure 1A) in female mice. The results exhibited that continuous VCD exposure induced significant ovarian damage on day 60, indicated by a decrease in ovarian weight (Mann–Whitney test, ** *p* < 0.01, Figure 1B) and a depletion of total follicles and mature follicles (two-tailed Student’s *t*-test, total: *t*_(*n* = 6)_= 2.297, *p* < 0.01; mature: *t*_(*n* = 6)_= 3.157, * *p* < 0.05; Figure 1C–E) in female mice. Simultaneously, radioimmunoassay showed that the serum progesterone level of VCD-treated mice also decreased significantly (Mann–Whitney test, * *p* < 0.05, Figure 1G) while the serum estradiol level remained unchanged (Mann–Whitney test, *p* = 0.16320, Figure 1F). In addition, estrous cycle patterns were monitored in the VCD-treated and control mice by daily vaginal lavage from day 55 to day 65. The results showed that the estrous cycle of VCD mice was disordered: the diestrus phase was significantly prolonged and the estrus phase was significantly shortened (Pearson’s chi-square test, Estrus: F _(*n* = 6)_ = 0.22, * *p* <0.05; Diestrus: F _(*n* = 6)_ = 0.22, * *p* <0.05, Figure 1H). In short, the results from mice suggested that on the 60th day, the VCD mice had already experienced significant ovarian dysfunction, which effectively simulated the pathophysiological functions in clinical menopausal women.

In addition to determination of VCD-induced ovarian damage in mice, we also examined behavioral phenotypes in VCD-treated mice on the 15th, 30th, 60th and 160th days, respectively (Figure 1A). The VCD mice showed behavioral despair from the 15th day, indicated by the increased immobility time in FST, and this depressive phenotype lasted until the 160th day (Mann–Whitney test, D15: *** *p* < 0.001, D30: *** *p* < 0.001, D60: ** *p* < 0.01, D160: * *p* < 0.05, Figure 1I). The reduction in exploration behavior (indicated by the decreased rearing frequency in OFT) also appeared on the 15th day, and the phenotype was maintained until the 160th day (Mann–Whitney test, D15: * *p* < 0.05, D30: ** *p* < 0.01, D60: * *p* < 0.05, D160: ** *p* < 0.01, Figure 1J). The anxiety phenotype appeared from the 30th day and lasted until the 160th day, exhibited by the decreased time spent in open arms of EPM (Sidak’s multiple comparisons test, *t* _(*n* = 6–12)_ = 2.953, * *p* < 0.05, ** *p* < 0.01, Figure 1K). Therefore, in the next experiments, the 60th day was selected as the time-point for detecting the behavioral and pathophysiological phenotypes of VCD mice and evaluating the potential therapeutic effects of kaempferol on menopausal depression.

### 3.2. Kaempferol Exhibits Antidepressant- and Anxiolytic-like Effects in Two Mice Models of Menopausal Depression (Figure 2)

The stresses of free radicals and antioxidant deficiencies are well known, both of which play a role in the pathogenesis of menopause [40]. Hence, we further explored the biological mechanisms underlying the potential therapeutic action of kaempferol, a flavonoid antioxidant on menopausal depression. Chronic treatment with kaempferol (10mg/kg, i.p., q.o.d., 14 days, from day 46-day 59, Figure 2A) exhibited a remarkable antidepressant- and anxiolytic-like effects in VCD mice (Figure 2B–F). Kaempferol can significantly decrease the immobility time of VCD mice in FST (Kruskal–Wallis test with Dunn’s multiple comparisons test, * *p* < 0.05, Figure 2B), increase the rearing frequency (Kruskal–Wallis test with Dunn’s multiple comparisons test, ** *p* < 0.01, *** *p* < 0.001, Figure 2C) in OFT and also significantly increase the time spent in open arms of EPM (one-way ANOVA and Tukey’s multiple comparisons test, F_(2, 31)_ = 5.012, * *p* < 0.05, Figure 2E). However, there was no significant change in the time spent in the central area of the open field (one-way ANOVA and Tukey’s multiple comparisons test, F_(2, 31)_ = 2.311, *p* = 0.188, Figure 2D,F).

To further confirm the antidepressant action of kaempferol in menopausal depression, we utilized another mice model of menopausal depression, developed in aged mice (11-month-old 129s1 mice) by exposing them to chronic unpredictable mild stress (CUMS) for 8 weeks (Figure 2G). In this experiment, kaempferol (10mg/kg, i.p., q.o.d., 14 days, from day 43 to day 64, Figure 2G) also exhibited antidepressant- and anxiolytic-like effects in CUMS-exposed aged mice (Figure 2H–K). Overall, the results suggested that kaempferol had beneficial therapeutic effects on menopausal depression.

### 3.3. Kaempferol Alleviates Hippocampal Oxidative Stress and Increases the Deacetylation of SOD2 and the Protein Level of Sirt3 (Figure 3)

**Figure 3:**
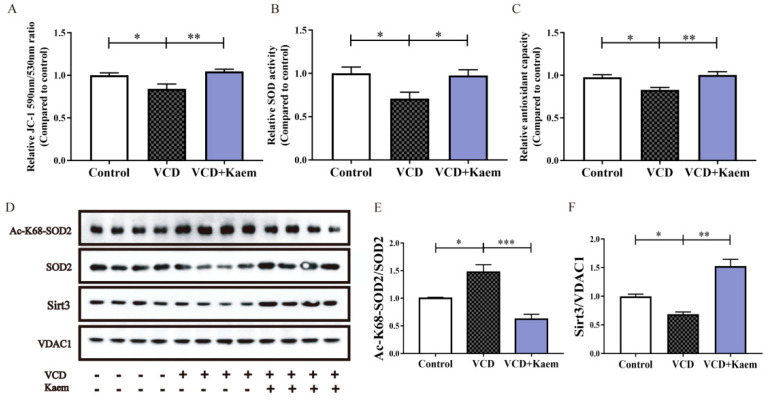
Kaempferol alleviates hippocampal oxidative stress and increases the deacetylation of SOD2 and the protein level of Sirt3. (**A**) Normalized statistic results of mitochondrial membrane potential in the hippocampus from all groups determined by the ratio between the fluorescence intensity at 590 nm (red fluorescence of polymer) and 530 nm (green fluorescence of monomer) after JC-1 staining. (**B**). Statistic result of superoxide dismutase (SOD) activity. (**C**). Normalized statistic results of mitochondrial total antioxidant capability. (**D**). Representative immunoblot of protein levels of Ac-K68-SOD2, SOD2 and Sirt3 in the hippocampal mitochondria from mice in three experiment groups. (**E**,**F**). SOD2-K68 acetylation (**E**) and Sirt3 (**F**) levels were normalized by SOD2 and VDAC1 respectively. Data are represented as mean ± SEM, *n* = 8–10 per group, one-way ANOVA analysis with Tukey’s multiple comparisons test:* *p* < 0.05, ** *p* < 0.01, *** *p* < 0.001.

As mentioned above, menopause is closely related with oxidative stress, mitochondrial dysfunction and damage [41]. As an active site of cellular redox homeostasis, mitochondria are also a major source of ROS in neurons. It is not surprising that maintaining the homeostasis and functions of this organelle is vital for neurons. In order to further explore the likely mechanism underlying the antidepressant action of kaempferol, we investigated mitochondrial parameters and oxidative stress markers after kaempferol treatment in mice. The results showed that kaempferol could cause a significant increase in mitochondrial membrane potential (one-way ANOVA and Tukey’s multiple comparisons test, F_(2, 25)_ = 7, ** *p* < 0.01, Figure 3A), total SOD activity (one-way ANOVA and Tukey’s multiple comparisons test, F_(2, 25)_ = 5.054, * *p* < 0.05, Figure 3B), total antioxidant capacity (one-way ANOVA and Tukey’s multiple comparisons test, F_(2, 25)_ = 7.963, ** *p* < 0.01, Figure 3C) in the hippocampus of VCD-treated mice.

We further investigated whether kaempferol exhibited antioxidant effects via increasing the function of endogenous antioxidant enzymes. The above-mentioned results showed that kaempferol could upregulate the SOD activity (Figure 3B). As we know, SOD2, the main SOD in mitochondria is activated by deacetylation. Therefore, we further tested the level of acetylation of mitochondrial proteins in the hippocampus after kaempferol treatment. The results showed that the acetylation of SOD2 in the hippocampal mitochondria was decreased after kaempferol treatment (one-way ANOVA and Tukey’s multiple comparisons test, F_(2, 9)_ = 20.73, *** *p* < 0.001, Figure 3D,E). Moreover, kaempferol treatment also increased the protein level of Sirt3 in the hippocampal mitochondria of VCD-treated mice, which shared a number of similarities with previous study (one-way ANOVA and Tukey’s multiple comparisons test, F_(2, 9)_ = 32.97, *** *p* < 0.001, Figure 3D,F).

### 3.4. Sirt3 Depletion Increases SOD2 Acetylation and Partially Prevents the Antidepressant- and Anxiolytic-like Effects as Well as Antioxidant Effects of Kaempferol (Figure 4)

**Figure 4:**
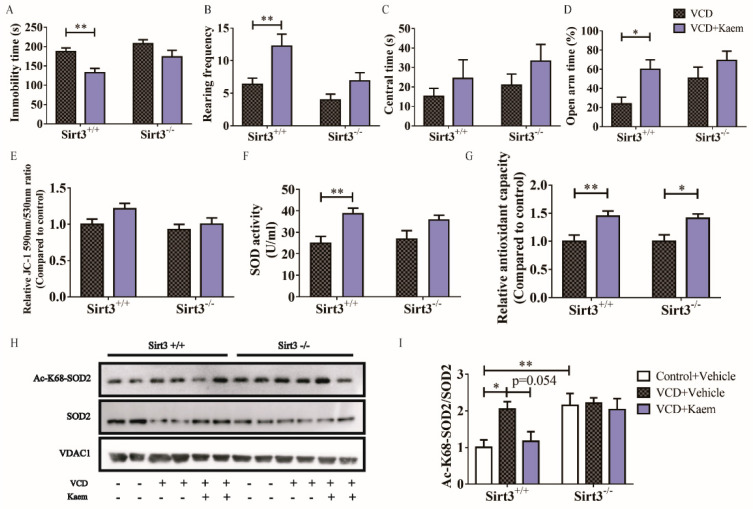
*Sirt3* depletion increases SOD2 acetylation and partially prevents antidepressant-, anxiolytic-like effects as well as antioxidant effects. (**A**). Immobility time in FST. (**B**,**C**) Rearing frequency (**B**) and time spent in the central area (**C**) in OFT. (**D**) Percentage of time spent in the open arms in EPM. (**E**–**G**) the membrane potential and antioxidant activity of mitochondria isolated from mouse hippocampal tissue of all experiment groups. Statistic results of mitochondrial membrane potential (**E**) were determined by the ratio between the fluorescence intensity at 590 nm (red fluorescence of polymer) and 530 nm (green fluorescence of monomer) after JC-1 staining. The antioxidant activity of mitochondria was reflected by the statistic result of SOD activity (**F**) and normalized statistic results of total antioxidant capability (**G**). (**H**) Representative immunoblot of protein levels of Ac-K68-SOD2 and SOD2 in the hippocampal mitochondria from mice in six experiment groups. (**I**) SOD2-K68 acetylation was normalized by SOD2. *n* = 12 per group in (**A**–**G**), *n* = 4 per group in (**I**) Data are represented as mean ± SEM. Two-way ANOVA analysis with Sidak’s multiple comparisons test or Mann–Whitney test: * *p* < 0.05, ** *p* < 0.01.

In order to further study whether Sirt3 mediate the beneficial therapeutic effects of kaempferol on menopausal depression, we further assessed the effects of kaempferol on VCD-treated *Sirt3*^−/−^ mice. *Sirt3* depletion did not aggravate the depression and anxiety phenotype and hippocampal oxidant status induced by VCD, but partially abrogated the antidepressant and anxiolytic effects of kaempferol (Figure 4A–D), as well as the antioxidant effects (Figure 4E–G). Interestingly, kaempferol still increased the total antioxidant capacity in *Sirt3*^−/−^ mice (two-way ANOVA and Sidak’s multiple comparisons test, F_(1, 28)_ = 18.32, * *p* < 0.05, Figure 4G), suggesting that kaempferol may have other ways to affect the total antioxidant capacity of mitochondria. In addition, knockout of *Sirt3* induced a significant increase in acetylation of SOD2 (Figure 4H,I) as mentioned in a previous study [42]. Meanwhile, kaempferol could not decrease the acetylation of SOD2 in *Sirt3*^−/−^ mice anymore (Figure 4H,I).

### 3.5. Overexpressing Sirt3 Exhibits Antidepressant- and Anxiolytic-like Effects Similar to Kaempferol (Figure 5)

**Figure 5:**
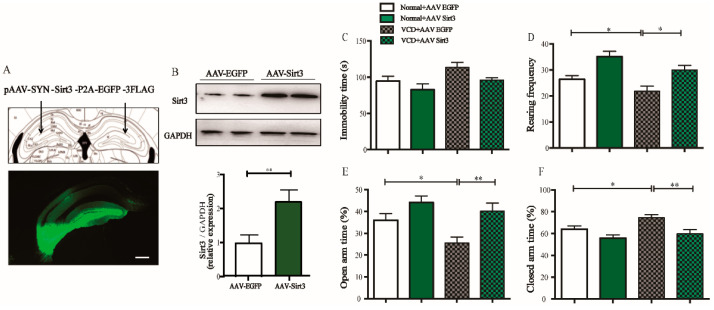
Overexpression of Sirt3 exhibits antidepressant- and anxiolytic-like effects similar to kaempferol. (**A**) The schematic diagram (upper panel) of the location for viral microinjection with a pAAV-SYN-Sirt3-P2A-eGFP-3FLAG (AAV-Sirt3) or a control pAAV-SYN-eGFP-3FLAG (AAV-eGFP) and fluorescence images (lower panel) showing efficient expression of AAV-Sirt3 vector in the hippocampus; Scale bar = 200 μm (*n* = 12–13 per group). (**B**) Representative images of Western blots (upper panel) and quantification of Sirt3 expression (lower panel) showing that AAV-Sirt3 injection increased Sirt3 expression in the hippocampus (*n* = 4 per group). (**C**) Immobility time in FST. (**D**) Rearing frequency in OFT. (**E**,**F**) Percentage of time spent in the open arms (**E**) and time spent in the closed arms in EPM. (**F**) Data are represented as mean ± SEM, two-way ANOVA analysis with Sidak’s multiple comparisons test: * *p* < 0.05, ** *p* < 0.01.

In order to further explore the relationship between Sirt3 and depression- and anxiety- like behaviors related menopause, we observed whether the overexpression of Sirt3 in the hippocampal neurons would affect the depression- or anxiety-like behaviors caused by VCD (Figure 5A). The results showed that overexpression of Sirt3 (Figure 5B) partially reversed the depression- and anxiety-like phenotypes induced by VCD, indicated by the increased rearing frequency in OFT (two-way ANOVA and Sidak’s multiple comparisons test, F_(1, 49)_ = 22.02,* *p* < 0.05, Figure 5D), and increased time in the open arm (two-way ANOVA and Sidak’s multiple comparisons test, F_(1, 49)_ = 13.00, ** *p* < 0.01, Figure 5E), decreased time in the closed arm of EPM (two-way ANOVA and Sidak’s multiple comparisons test, F_(1, 49)_ = 13.00, ** *p* < 0.01, Figure 5F) but the immobility time in the FST in VCD mice was not significantly changed (Figure 5C). These results proved that overexpression of Sirt3 effectively relieved anxiety symptoms caused by VCD.

### 3.6. Kaempferol Promotes ERRα Nuclear Translocation (Figure 6)

**Figure 6:**
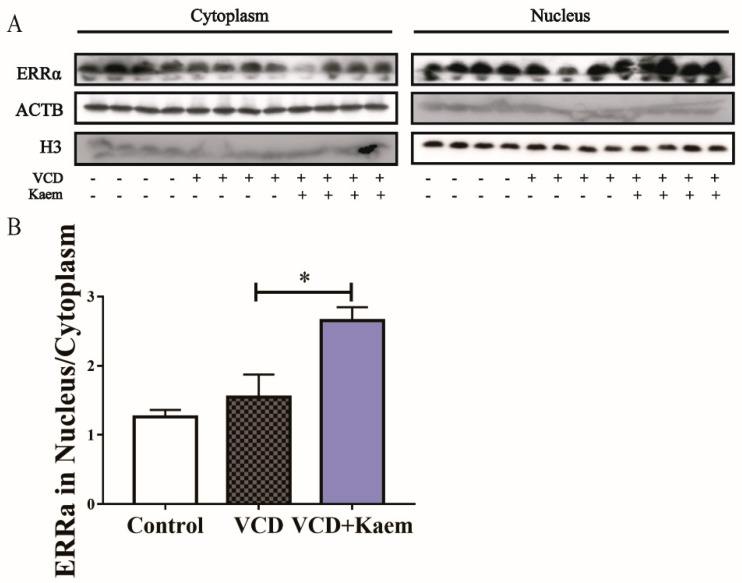
Kaempferol promotes nuclear entry of ERRα in mouse hippocampal. The nuclei and cytoplasm of hippocampus cells were isolated in the control group, VCD group and kaempferol treatment group. Then, the levels of ERRα protein in the nucleus and cytoplasm were tested (**A**,**B**). Ratio of ERRα in the nuclei and cytoplasm were calculated. One-way ANOVA analysis was used for Tukey’s multiple comparisons test. Data are represented as mean ± SEM, *n* = 8 per group; * *p* < 0.05.

We have made some preliminary attempts to capture the potential mechanism underlying the action of kaempferol on Sirt3 expression in vivo. The results showed that kaempferol could promote ERRα nuclear translocation (one-way ANOVA and Tukey’s multiple comparisons test, F_(2, 9)_ = 10.51, * *p* < 0.05, Figure 6), which hinted that kaempferol might regulate Sirt3 expression via promoting ERRα nuclear translocation. Admittedly, the precise mechanisms still need more research.

## 4. Discussion

In this study, we verified that kaempferol has potential beneficial effects on menopausal depression, which were assessed in VCD-induced rodent (mice and rat) models of menopause. Additionally, our study further uncovered that Sirt3, the major deacetylase in mitochondria, mediated the antioxidant and antidepressant effects of kaempferol. Moreover, kaempferol also promoted ERRα nuclear translocation.

Menopause is a special period for women; it might come with mood disturbance and general symptoms of anxiety and depression in addition to physical symptoms. To predict the post-treatment outcomes and to develop new therapeutic strategies, scientists established several rodent models of menopause, including natural reproductive senescence, ovariectomy and ovotoxins to induce accelerated ovarian failure [43]. Obviously, each model has its own advantages and disadvantages. Among the two classes of ovotoxins to induce accelerated ovarian failure, VCD is the best studied.

Although scientists have evaluated many of the system-level outcomes of ovarian hormone depletion in the VCD-induced animal model of menopause [44], the mood-related phenotypes have not been observed until recently [25]. Wang et al. evaluated the depression- and anxiety-like behaviors of VCD-treated mice at day 17 and day 18 after the cessation of VCD (i.p. 160 mg/kg, 20 consecutive days) [25]. They found that VCD significantly induced anxiety-like but not depression-like behaviors [25]. Conversely, our observations were longer and more detailed. However, we did not observe any significant mood-related phenotypes in FST, OFT and EPM at day 15 after VCD treatment (Figure 1I–K). VCD-treated mice exhibited partial depression- and anxiety-like phenotypes on day 30 after VCD exposure (Figure 1I–K). In addition, these phenotypes were more significant and continuous until day 60 after VCD exposure. However, at day 160 after VCD exposure, these phenotypes gradually faded. These results are in line with the previous studies hinting that chronic VCD exposure would induce significant depression- and anxiety-like phenotypes, followed by a slow recovery. So, in our opinion, day 60 after VCD exposure is a good time-point to study menopausal depression.

Kaempferol is well-known as a flavonoid antioxidant rich in fruits and vegetables and meanwhile has anti-inflammatory functions. Ten years ago, kaempferol was reported to act as a free radical scavenger with a potential role in preventing and treating oxidative stress [45]. However, kaempferol regulates the level of antioxidant enzymes in vivo and in vitro, such as catalase, glutathione peroxidase and glutathione-*S*-transferase [46,47]. Our results provide more cues about the molecular mechanisms of kaempferol in vivo, that it increased the activity of SOD2 via regulating the expression of Sirt3 (Figure 4), a mitochondrial deacetylase which activates SOD2 by deacetylation of specific lysine residues. Interestingly, in addition to antioxidant enzymes, scientists found that kaempferol strongly inhibited the overproduction of proinflammatory cytokines and LPS-stimulated NF-κB signaling pathways activation [48]. Moreover, the beneficial effects of kaempferol via modulating NF-kB by kaempferol have also been explored in several inflammatory disease models [49,50]. Although we did not observe the influence of kaempferol on inflammatory mediators, our results show that sirt3 partially mediated the antidepressant effects of kaempferol. According to these results, we suggest that anti-inflammatory and other antioxidant functions of kaempferol might also involve beneficial effects on depression.

Because of the anti-oxidant and anti-inflammatory effects of kaempferol, many scientists have raced to investigate its potential uses for the treatment of some inflammatory diseases. Some studies have suggested its beneficial effects in decreasing the risk of chronic diseases, especially inflammatory disorders [17] and CNS diseases [16]. Recently, depression has also been involved [51,52]. Gao and collaborators [51] provided evidence for the antidepressant effects of kaempferol, which might be mediated by its antioxidant and anti-inflammatory capacities and its effects on the AKT/β-catenin cascade in the prefrontal cortex. Garza et al. also observed an important antidepressant modulation of kaempferol on pregnant rats [52]. Additionally, our study provides some direct support for its potential beneficial effects on menopausal depression (Figure 2). We further propose Sirt3 as a promising molecular target for its antioxidant and antidepressant effects (Figure 3 and Figure 4).

Interestingly, a previous study has given some cues about the direct effects of kaempferol on Sirt3 [53]. The authors verified that in human embryonic kidney cells 293 (HEK 293) and mouse kidney proximal tubule epithelial (Tkpts) cells, kaempferol could augment mitochondrial localization of Sirt3 [53]. In another earlier study, Cimen et al. suggested that kaempferol stimulated Sirt3 expression in K562 cell lines [54]. These results in vitro were in line with our present results in vivo. Although they did not clarify how kaempferol regulate Sirt3 expression, we have made some preliminary attempts to capture the potential mechanisms.

As in brown adipocytes [55], Sirt3 is reported also to be regulated by ERRα and peroxisome proliferator-activated receptor gamma coactivator-1α (PGC-1α) in the dopaminergic neurons [56], and the ERRα/PGC1α-Sirt3 pathway plays a vital role in protecting against dopaminergic neuronal death. Meanwhile, kaempferol is shown to act as an inverse agonist for ERRα and ERRγ, binding to ERRα and ERRγ and blocking their interactions with coactivator PGC-1α [57]. However, knockdown of PGC-1α did not affect Sirt3 expression [58]. Our data in the present study hinted that kaempferol might increase the expression of Sirt3 via promoting ERRα nuclear translocation, which shed some light on the mechanism underlying the action of kaempferol on Sirt3 expression.

The study has potential limitations. One limitation is the smaller sample size (4–5 per group) in the experiment, when further demonstrating the antidepressant effect of kaempferol in aged female mice exposed to CUMS. If our study was based on a larger sample size, it may have generated more accurate results. Another limitation is that we only observed that kaempferol promoted the nuclear translocation of ERRα. Based on these data, we speculate that kaempferol might increase Sirt3 expression through ERRα. However, the precise mechanism needs further research. The third limitation is that we have neglected the assessment of anhedonia in rodent models of depression. This may limit a comprehensive assessment of the VCD-induced depression phenotype and the antidepressant effect of kaempferol.

## 5. Conclusions

In conclusion, we revealed an important mechanism by which antioxidant-mediated kaempferol functions in regulating mood in a VCD-induced rodent model of menopausal depression via upregulating Sirt3 expression and subsequently de-acetylating and activating SOD2. Moreover, kaempferol might increase the expression of Sirt3 via promoting ERRα nuclear translocation, which requires more widespread research. As such, the present findings hold important implications of kaempferol for menopausal depression treatment.

## Data Availability

The data presented in this study are available in the article and Supplementary Materials.

## Supplementary Materials

The following supporting information can be downloaded at: https://www.mdpi.com/article/10.3390/antiox11101886/s1, Table S1: the experimental design with the number and subdivision of animals and timing of sacrifice.
