# Supplementary material for: Sirtuin 3 Plays a Critical Role in the Antidepressant- and Anxiolytic-like Effects of Kaempferol
Source: Antioxidants (Basel). 2022 Sep 23;11(10):1886. doi: 10.3390/antiox11101886 (PMC9598871; doi:10.3390/antiox11101886)
Supplement: Supplementary file 1 [file antioxidants-11-01886-s001.zip › antioxidants-1837283-supplementary.pdf]

Table S1. The experimental design with the number and subdivision of animals and timing of sacrifice

		Time line					
Program	group	Step 1	Step 2	Step 3	Step 4	Step 5	Number of mice
Experiment 1: VCD induced ovary failure	1	VCD treatment for 14 days	Behavior test on Day 15	Sacrifice on Day 16			9
	2	Oil treatment for 14 days					6
	3	VCD treatment for 14 days	Behavior test on Day 30	Sacrifice on Day 31			7
	4	Oil treatment for 14 days					11
	5	VCD treatment for 14 days	Behavior test on Day 60	Blood taken, Sacrifice, Ovary taken on Day 61			7
	6	Oil treatment for 14 days					5
	7	VCD treatment for 14 days	Estrous cycle patterns monitoring on Day55-Day60	Behavior test on Day 160	Sacrifice on Day 161		6
	8	Oil treatment for 14 days					4
Experiment 2: Kaempferol treatment on VCD model	1	No treatment for 14 Days	No treatment for 31 Days	No treatment for 14 days	Behavior test on Day 60	Blood taken, Sacrifice, Ovary/Hippocampus taken on Day 61	13
	2	VCD treatment for 14 days	No treatment for 31 Days	No treatment for 14 days			11
	3	VCD treatment for 14 days	No treatment for 31 Days	Kaempferol treatment for 14 days			10

Experiment 3: Kaempferol treatment on aged mice exposed to CUMS	1	Female Mice grow up to 11 month old	No treatment for 42 Days	No treatment for 14 Days	Behavior test on Day 57	Sacrifice	6
	2		CUMS treatment for 42 Days	CUMS treatment for 14 Days			4
	3		CUMS treatment for 42 Days	CUMS and kaempferol treatment for 14 Days			5
Experiment 4: Kaempferol treatment on VCD model in Sirt3-KO mice	1	WT	VCD treatment for 14 days	No treatment for 32 Days	No treatment for 14 days	Behavior test on Day 60, Sacrifice, Hippocampus taken on Day 61	11
	2	Sirt3-KO	VCD treatment for 14 days	No treatment for 32 Days	No treatment for 14 days		11
	3	WT	VCD treatment for 14 days	No treatment for 32 Days	Kaempferol treatment for 14 days		11
	4	Sirt3-KO	VCD treatment for 14 days	No treatment for 32 Days	Kaempferol treatment for 14 days		10
Experiment 5: Sirt3 over expression on VCD model	1	VCD treatment for 14 days	No treatment for 32 Days	AAV-EGFP injection	No treatment for 14 days	Behavior test on Day 60, Sacrifice, Hippocampus taken on Day 61	12
	2	VCD treatment for 14 days	No treatment for 32 Days	AAV-Sirt3-OE injection	No treatment for 14 days		13
	3	VCD treatment for 14 days	No treatment for 32 Days	AAV-EGFP injection	No treatment for 14 days		12
	4	VCD treatment for 14 days	No treatment for 32 Days	AAV-Sirt3-OE injection	No treatment for 14 days		13
